# Hair cortisol of pigs in mixed organic farms: the influence of season, breeding system and sex

**DOI:** 10.3389/fvets.2024.1491785

**Published:** 2024-12-13

**Authors:** Eva Nadlučnik, Tilen Vake, Ana Šket, Ana Žižek, Tomaž Snoj, Marina Štukelj

**Affiliations:** ^1^Clinic for Ruminants and Pigs, Clinic for Reproduction and Large Animals, Veterinary Faculty, University of Ljubljana, Ljubljana, Slovenia; ^2^Institute of Preclinical Sciences, Veterinary Faculty, University of Ljubljana, Ljubljana, Slovenia; ^3^Veterinary Faculty, University of Ljubljana, Ljubljana, Slovenia

**Keywords:** hair cortisol, organic farming, pigs, Krškopolje, animal welfare, HPA axis

## Abstract

**Introduction:**

Measurement of hair cortisol concentration (HCC) is a useful tool for assessing the activity of the hypothalamic-pituitary-adrenal axis and thus evaluating the long-term adrenocortical response in different animal species and breeds. Robust indigenous pig breeds are highly adapted to the local environment and are preferred for organic farming, compared to the commercial breeds. We investigated whether seasonality, breeding system (indoor or outdoor) and sex influence HCC of pigs reared on organic farms.

**Materials and methods:**

Fifty-three pigs of the indigenous Slovenian Krškopolje breed were divided into three groups. Group Ind (*n* = 15) was housed indoors, groups Out-1 (*n* = 18) and Out-2 (*n* = 20) were housed outdoors on two different farms for the duration of 9 months. Hair was sampled once per season in the withers area of each pig and HCC was determined using a previously validated ELISA.

**Results and discussion:**

The effect of seasonality was found to be significant and more pronounced in pigs reared outdoors. HCC were highest and varied most in winter for all groups, while they were lowest and varied less in summer and autumn. The highest HCC was measured in group Out-1 in winter, as it was the only group housed outdoors at that time. Group Ind had significantly higher HCC in summer compared to the groups Out-1 and Out-2, which could be due to hair cortisol degradation by the UV light exposure in outdoor groups. Sex had no effect on HCC. Our study suggests that seasonality and housing type influence the HCC of pigs.

## 1 Introduction

Modern pig farming is characterized by intensive indoor breeding systems in which pigs live in an impoverished environment, are exposed to many stress factors and their general welfare is often low (1–4). Organic pig production systems combine good environmental practices with high animal welfare standards (5). On organic farms, pigs are usually kept in one of the following three systems: (A) all pigs kept indoors with permanent access to the outdoors, (B) all pigs kept outdoors with shelters or (C) combined systems where some categories of pigs are kept indoors and others outdoors, e.g., pregnant sows outdoors and lactating sows indoors (6). The use of indigenous breeds is preferred in organic farming as they can adapt to local conditions without any negative impact on animal welfare (5). The only Slovenian indigenous pig breed is the Krškopolje pig, which is known for its robustness as it can adapt to a variety of environmental conditions (7). Pigs in outdoor systems can express natural behavior (e.g., rooting, grazing, foraging). Consequently, fewer stereotypies, tail biting and injuries are observed, which is linked to improved welfare (8, 9). On the other hand, they require higher feed intake due to increased activity and thermoregulation, especially in winter (9), and possible contact with wild animals makes them susceptible to diseases such as African swine fever (10).

Animal welfare can be assessed using protocols and questionnaires (11, 12), by observing animal behavior (13), by measuring vocalization (14) or by measuring stress biomarkers in body fluids (15–17). Cortisol and its metabolites are useful biomarkers that provide information about the physiological state of the animal and insight into the activity of the hypothalamic-pituitary-adrenal (HPA) axis. The biomarkers can be determined in serum, saliva, urine, feces, milk and hair (18). During hair growth, cortisol is continuously loaded into the hair shaft. Therefore, using hair as a sample for the detection of cortisol can provide information about the activity of the HPA axis over a period of weeks to months, depending on hair length and growth rate (19). Hair sampling is minimally invasive and painless (20), and the samples remain stable over longer periods of time (21).

The aim of our study was to determine whether seasonality, breeding system (indoor, outdoor) and sex influence the hair cortisol concentration (HCC) in pigs, reared on organic farms.

## 2 Materials and methods

### 2.1 Ethics approval

This study was carried out as a part of the ERA-Net CORE Organic Cofound project—Robust animals in sustainable mixed free-range systems project (ROAM-FREE) and was ethically approved by the Ministry of agriculture, forestry, and food (U34401-6/2022/11). The overall objective of the ROAM-FREE project is to investigate how mixed free range production systems can improve animal welfare, environmental and economic sustainability, and biodiversity in organic pig farming. The pigs were kept in accordance with Regulation (EU) 2018/848 on organic production (5) and national animal welfare laws and regulations (22). The welfare and health of the pigs were regularly assessed by a farm veterinarian.

### 2.2 Animals and housing

The study was conducted on 53 pigs of the Krškopolje breed: 27 females and 26 castrated males. All pigs originated from the same Slovenian organic pig farm. The farm had a free-farrowing system and straw-bedded pens, with outdoor access for growing pigs. Tail docking or teeth clipping was not performed, as they did not have problems with tail biting. The male pigs were surgically castrated at the age of 4–6 days with the use of analgesia (Metacam, Boehringer Ingelheim, Germany; dosed at 0.4 mg/kg.). The pigs were purchased at 8 weeks of age and then divided into three farms, all located in the Littoral-Inner Carniola region in southwestern Slovenia. Pigs from the same litters were relocated to the same group to keep the groups as stable as possible. To assess the ambient temperatures and solar exposure, we collected the measurements from meteorological station, nearest to the farms (23). Solar exposure was measured as hours of direct sunlight per month.

#### 2.2.1 Group Out-1

**Figure 1:**
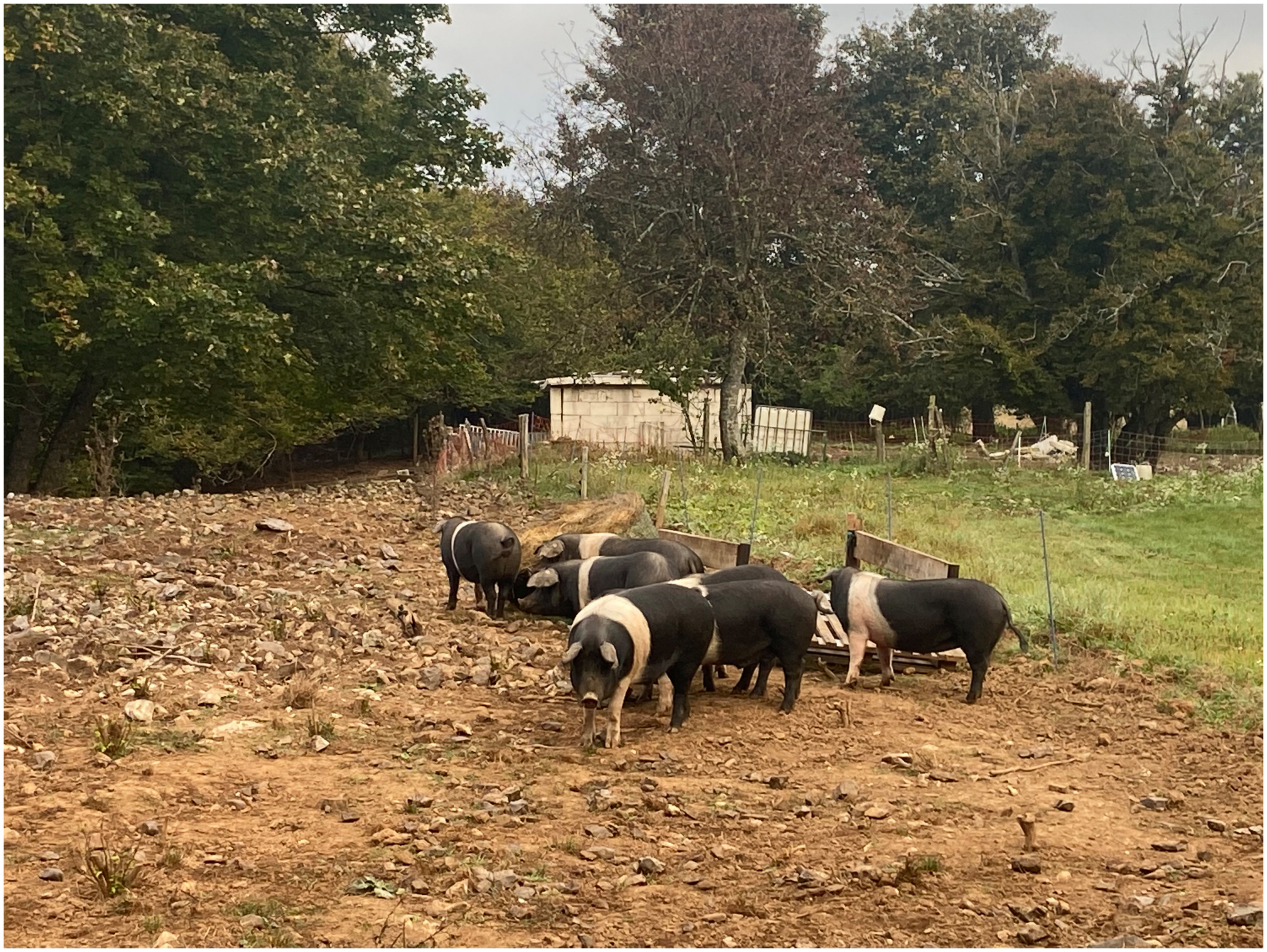
Pigs from group Out-1 on a large pasture, shaded area with trees and shelter visible.

The first group of pigs (Out-1) (*n* = 18, 8 males, 10 females) was reared outdoors on a large grass pasture (12,600 m^2^) (Figure 1). The pasture was partly covered with trees (on the area of 5,400 m^2^) and had one covered shelter (41 m^2^). The shelter was bedded with straw and had one water trough and one feed trough inside. The pigs were housed outdoors throughout the year—in all seasons and sampling days.

#### 2.2.2 Group Out-2

**Figure 2:**
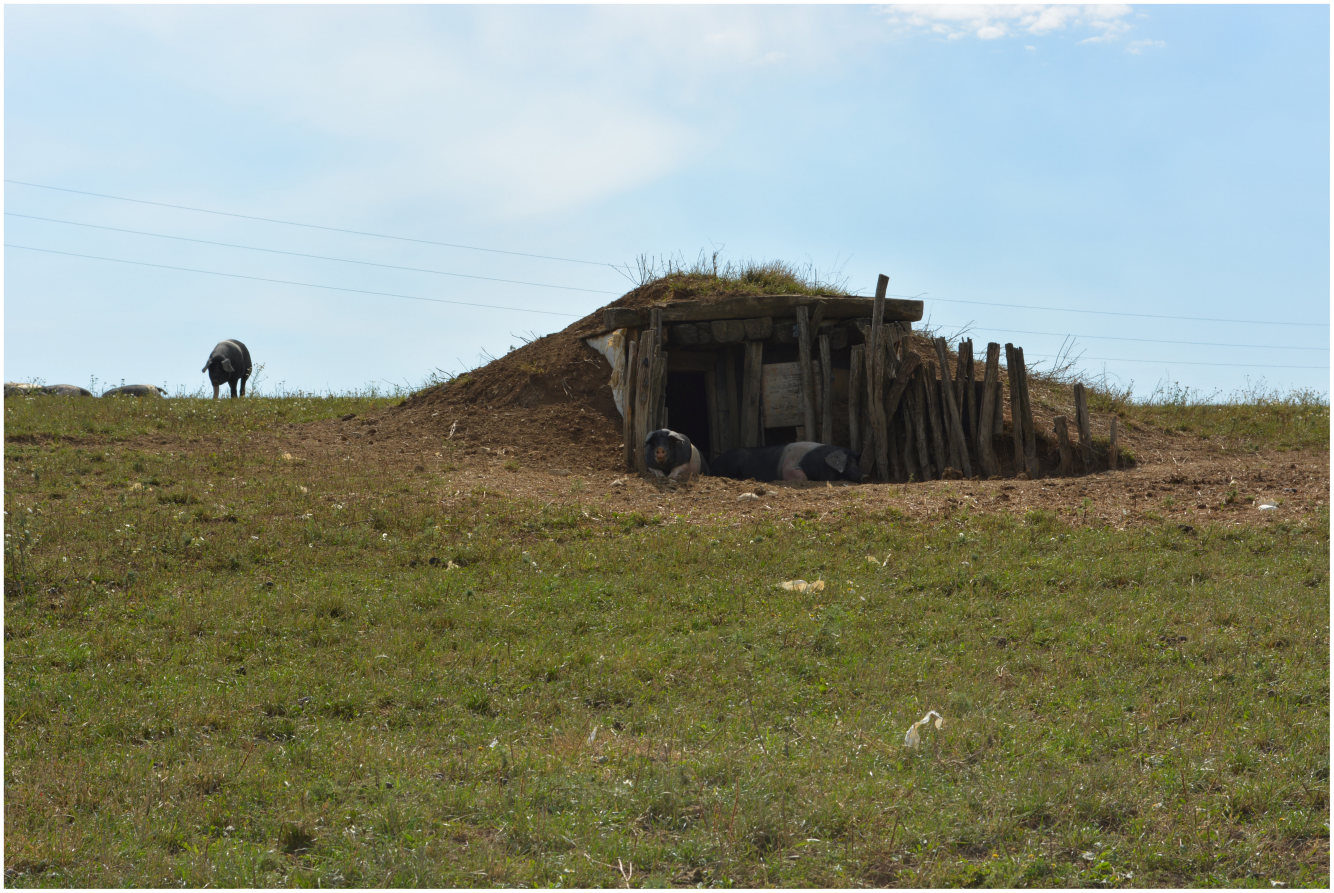
Pigs of group Out-2 on a large pasture, one dugout visible.

The second group of pigs (Out-2) (*n* = 20, eight males, 12 females) was reared outdoors on a large grass pasture (9,000 m^2^) (Figure 2). There were no trees on the pasture, but there were two straw bedded dugouts (13.5 m^2^ each). They were big enough to house all the pigs and offered additional shade in the entrance of the dugouts. Nipple drinker and feeder was provided. In winter, the Out-2 group was housed indoors in a large pen (2.5 m^2^ floor area per pig) bedded with deep straw bedding, as this was the farm's practice. The pen was in a closed but unheated barn, that was separate from other animals present on the farm. The barn was directly next to the pasture, so when the pigs were let out in spring (March), no relocation was necessary. The pigs remained on a pasture until the end of our study.

#### 2.2.3 Group Ind

**Figure 3:**
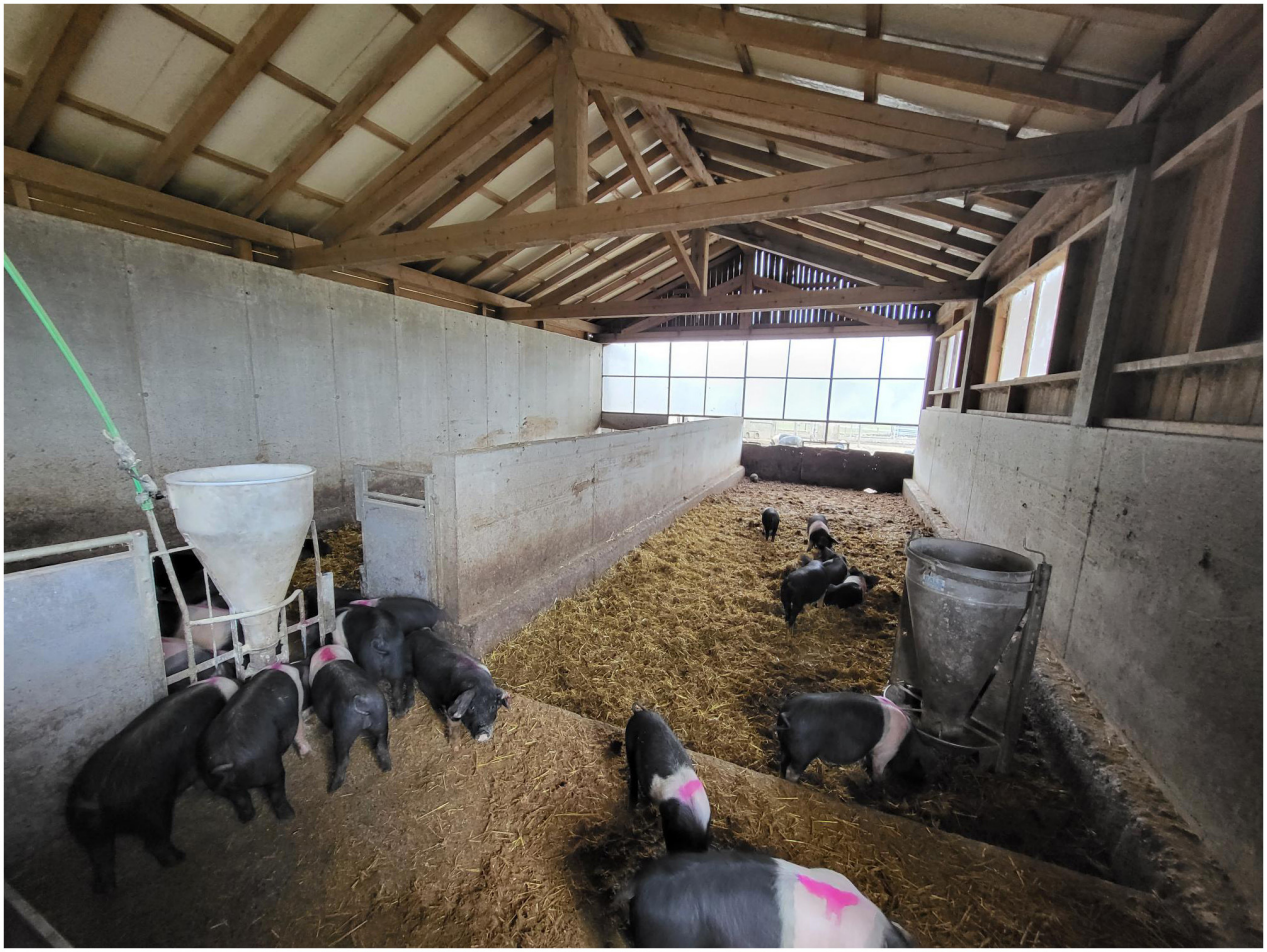
Pigs of group Ind in the large barn.

Pigs in the third group (Ind) (*n* = 15, eight males, seven females) were reared indoors all year round (Figure 3). They were housed in a large bright barn (50 m^2^, 2.5 m^2^ floor area per pig), lined with straw. The barn had high concrete walls and several windows. Ventilation was natural—open windows. The barn had no heating. Nipple drinkers and feeders were provided. No other animals were present in the barn.

All pigs were fed an organic diet consisting of 60% barley, 30% wheat and 10% sunflower meal. The pigs in the Out-1 group were fed twice a day: 35 kg of feed in the morning and 15 kg of feed in the afternoon. The Out-2 and Ind groups of pigs were fed *ad libitum*.

### 2.3 Study design

The study was conducted over a period of 9 months, from February 2023 to October 2023. The pigs' hair was collected on four sampling days in winter (February), spring (May), summer (July), and autumn (October)—four hair samples were taken from each pig. In total, 212 hair samples were obtained. To assess the differences in HCC during winter sampling, pigs' hair was collected 3 weeks after their relocation. This timing was selected to allow an adjustment period, as the pigs initially lived in the same environment during the first weeks of life, ensuring that the HCC reflected conditions post-relocation. Krškopolje pigs are black and white, with a white to pink belt running across the chest and forelimbs. Hair was collected from the withers area, regardless of hair color (black or white), to minimize the influence of the sampling region on the HCC. Hair was collected from different sites of the withers area. Based on our experience and observations, this area tends to remain clean, which supports the accuracy of cortisol measurements by minimizing potential external contamination influences (24). On sampling days, we did not notice any contamination in this area. The hair samples were ~3 cm long and we always used the whole hair for further processing. Samples were collected between 9:00 and 11:00 a.m., to minimize the influence of circadian rhythm on HCC. However, ultradian and seasonal rhythms can still affect hair cortisol concentrations (25). After collection, the hair was stored in tightly sealed plastic bags at 20°C until further analysis.

### 2.4 Hair sample preparation

The extraction of cortisol from hair samples was performed as described by Nedić et al. (26) with slight modifications. From each pig, we obtained ~1 g of whole native hair. Approximately 0.2 g of this whole native hair sample was randomly selected and placed in a mortar and about 20 mL of liquid nitrogen was added and left there until it had evaporated. The frozen hair was ground with a pestle to minimize the influence of hair color (we collected both black and white hair) and to minimize the influence of different cortisol concentrations in different hair segments. The hair powder was then dried at 40°C for 30 min. We weighed 50 mg of the hair powder and placed it in a polypropylene tube. Three milliliters of 80% methanol were added to the tube and shaken at 400 rpm for 18 h at room temperature. The tubes were then centrifuged at 2,500 × g for 15 min and the supernatant was transferred to a new tube. The supernatant was dried under the nitrogen stream at 40°C in the evaporator. In addition, 0.8 mL of 80% methanol was added to each sample and vortexed for 1 min. The samples were then centrifuged at 2,500 × g for 15 min. The extract was carefully pipetted off and added to the first extract, which was dried under a stream of nitrogen. The dried samples were stored at −20°C until analysis. One hour before measuring the HCC, the dried samples were reconstituted in 500 μL PBS.

### 2.5 Cortisol analysis

HCC was determined with a commercial Cortisol ELISA (DES6611, Demeditec, Kiel, Germany) according to the instructions for use. Absorbance was measured with a Multiskan FC microplate photometer (Thermo Fisher Scientific, Waltham, USA) at 450 nm. The results obtained in ng cortisol per mL of extract were converted to ng cortisol per g of hair. A partial validation of the ELISA kit, which included the determination of the intra-assay and inter-assay coefficients of variation, was evaluated. Samples of hair extracts with low and high cortisol concentrations were run 20 times in one ELISA and repeated in triplicate in the next ELISA. The intra- and inter-assay coefficients of variation were 7.1 and 8.9% for high (76.4 ng/g) and 12.2 and 13.9% for low (23.1 ng/g) HCC, respectively.

Data on the specificity of the method was provided by the manufacturer of the ELISA kit. Thus, the cross-reactivity with other steroids was as follows (in %): prednisolone 100, 11-deoxycortisol 50, corticosterone 6.2, 11-deoxycorticosterone 2.6, 17-hydroxyprogesterone 1.3, prednisone 0.9, cortisone 0.8, estrone 0.1 and <0.1 for androstenedione, testosterone, dexamethasone, estriol, estradiol, progesterone, pregnenolone and danazol.

### 2.6 Statistical analysis

The statistical analysis was performed with RStudio (version 2023.12.1), employing *lme4* and *emmeans* packages for data analysis and *ggplot2* for data visualizations. A generalized linear mixed model (GLMM) was fitted with a gamma family distribution and a log-link function to the HCC. Fixed effects included sex and a two-way interaction between breeding system (group) and season, while animal ID was included as a random intercept to account for repeated measures across individuals. The model formula was constructed as follows:


$$\begin{array}{l} HCC\;\sim \;Group\;\times \;Season+Sex+\left(1\;|\;ID\right) \end{array}$$

A total of 198 observations from 53 unique animal IDs were analyzed, with the animal ID serving as a random effect cluster representing the hierarchical structure of the data. Animal age was not included as a covariate in the model because all subjects began the study at the same age and aged uniformly with each season, so that seasonal changes inherently accounted for age progression. To assess model fit, the GLMM was also compared with a simplified generalized linear model (GLM) without the random effect term. The GLMM showed a significantly better fit based on lower values of Akaike Information Criterion (AIC) and the Bayesian Information Criterion (BIC). The GLMM model estimates were then used to compare the HCC of the different groups within each season and to compare the HCC change over successive seasons within each group. The Tukey's correction was applied to the pairwise comparisons of group estimates within each season, while the Sidak's correction was used for comparisons of successive seasons within each group, both performed on the response scale for ease of interpretation. Reported HCC differences and ratios thus reflect model-based predictions of mean HCC under specified conditions. The significance level was set at *p* < 0.05. To validate the modeling assumptions, we examined deviance and Pearson residuals, which confirmed the suitability of the gamma family and log link in this context. The Nakagawa method was used to quantify variance explained, providing both marginal *R*^2^ (variance explained by fixed effects) and conditional *R*^2^ (total variance explained by both fixed and random effects).

## 3 Results

**Figure 4:**
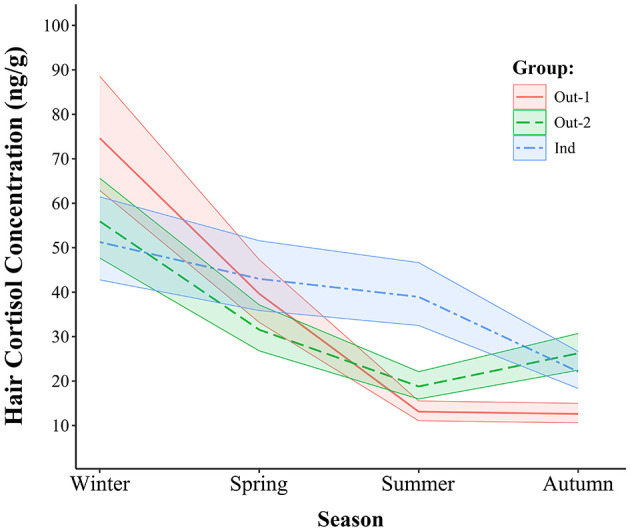
Estimated mean hair cortisol concentrations with 95% confidence intervals across all groups and seasons.

A summary of model fit statistics, variance explained, random effects, and fixed effects of the GLMM can be found in Table 1. The GLMM was selected as the final model based on a comparison with a simplified GLM, where it showed a better fit, as evidenced by lower AIC and BIC values (GLMM: AIC = 1,471.4, BIC = 1,520.7) compared to the GLM (AIC = 1,492.7, BIC = 1,538.8). The GLMM also achieved a log-likelihood of −720.7 and a deviance of 1,441.4. The conditional *R*^2^ and marginal *R*^2^ of the model indicate that 79.7% of the variance in HCC was explained by both the fixed and random effects, with 74.8% explained by the fixed effects alone. The random effects showed that the random intercept for animal ID contributed a variance of 0.01993 with a standard deviation of 0.1412, while the residual variance was 0.08476 with a standard deviation of 0.2911, reflecting some individual variability in HCC. The fixed effects analysis in the type II ANOVA table shows significant influences of group, season, and the interaction between group and season on HCC. In particular, group (χ^2^ = 14.058, df = 2, *p* < 0.001), season (χ^2^ = 510.650, df = 3, *p* < 0.001), and the group-by-season interaction (χ^2^ = 165.420, df = 6, *p* < 0.001) all had statistically significant effects. The effect of sex was not statistically significant (χ^2^ = 1.208, df = 1, *p* = 0.242). The model predictions of the estimated HCC means are shown in Figure 4 and the pairwise comparisons within groups and within seasons of interest are presented in Table 2.

**Table 1:** Model fit, variance explained, and fixed effects summary for generalized linear mixed effects model on hair cortisol concentrations in pigs.

**Model fit statistics**				
**AIC**	**BIC**	**Log-Likelihood**	**Deviance**	
1,471.4	1,520.7	−720.7	1,441.4	
**Variance explained**				
**Conditional** *R*^2^		**Marginal R** ^2^		
0.797			0.748	
**Random effects**				
**Random effect**		**Variance**	**Std. deviation**	
ID		0.01993	0.1412	
Residual		0.08476	0.2911	
**Type II ANOVA table of fixed effects**				
**Effect**	**Chi-squared**	**Df**	***p*** **-value**	**Significance**
Group	14.058	2	<0.001	^***^
Season	510.650	3	<0.001	^***^
Sex	1.208	1	0.242	
Group : season	165.420	6	<0.001	^***^

AIC, Akaike Information Criterion; BIC, Bayesian Information Criterion; Df, Degrees of freedom.

Significance codes: “^***^” < 0.001.

**Table 2:** Seasonal and group comparisons of model-estimated means of hair cortisol concentrations in pigs (back-transformed from the log to the linear scale).

**Contrast**	**Group**	**Ratio**	**Std. error**	**Lower CI**	**Upper CI**	***z*-statistic**	***p*-value**	**Significance**
**Seasonal comparisons within groups**								
Winter/Spring	Out-1	1.883	0.191	1.479	2.398	6.255	<0.001	^***^
Spring/Summer		3.022	0.301	2.383	3.833	11.115	<0.001	^***^
Summer/Autumn		1.040	0.101	0.825	1.311	0.407	0.968	
Winter/Spring	Out-2	1.773	0.164	1.422	2.210	6.199	<0.001	^***^
Spring/Summer		1.679	0.158	1.342	2.101	5.523	<0.001	^***^
Summer/Autumn		0.716	0.065	0.576	0.890	−3.664	0.001	^**^
Winter/Spring	Ind	1.192	0.122	0.933	1.523	1.712	0.239	
Spring/Summer		1.104	0.112	0.867	1.407	0.979	0.696	
Summer/Autumn		1.761	0.184	1.372	2.260	5.416	<0.001	^***^
**Contrast**	**Group**	**Ratio**	**Std. error**	**Lower CI**	**Upper CI**	***z*** **-statistic**	***p*** **-value**	**Significance**
**Group comparison within seasons**								
Out-1/Out-2	Winter	1.334	0.158	1.009	1.763	2.426	0.040	^*^
Out-1/Ind		1.456	0.183	1.083	1.957	2.976	0.008	^**^
Out-2/Ind		1.091	0.133	0.818	1.454	0.711	0.756	
Out-1/Out-2	Spring	1.256	0.153	0.943	1.672	1.867	0.148	
Out-1/Ind		0.921	0.118	0.682	1.245	**–**0.634	0.801	
Out-2/Ind		0.733	0.091	0.549	0.980	**–**2.501	0.033	^*^
Out-1/Out-2	Summer	0.698	0.083	0.528	0.922	**–**3.023	0.007	^**^
Out-1/Ind		0.336	0.042	0.251	0.451	**–**8.703	<0.001	^***^
Out-2/Ind		0.482	0.059	0.361	0.644	**–**5.916	<0.001	^***^
Out-1/Out-2	Autumn	0.480	0.056	0.363	0.634	**–**6.188	<0.001	^***^
Out-1/Ind		0.570	0.073	0.422	0.770	**–**4.376	<0.001	^***^
Out-2/Ind		1.186	0.146	0.888	1.585	1.386	0.347	

CI, 95% confidence interval.

Significance codes – “^***^” < 0.001, “^**^” < 0.01, “^*^” < 0.05.

### 3.1 Comparisons of seasonal cortisol concentrations within each group

Comparisons of seasonal HCC within each group showed significant seasonal fluctuations in the Out-1 and Out-2 groups but more limited variation in the Ind group. In Out-1, HCC were 88.3% greater in winter than in spring (*p* < 0.001), and 202.2% greater in spring than in summer (*p* < 0.001). However, no significant difference was observed between summer and autumn. In the Out-2 group, concentrations in winter were 77.3% greater compared to those in spring (*p* < 0.001) and concentrations in spring were 67.9% greater compared to those in summer (*p* < 0.001). In addition, a significant increase of 39.7% from summer to autumn was observed in this group (*p* = 0.001). The seasonal differences were less pronounced in the Ind group. No significant differences were found between winter and spring or between spring and summer. However, HCC were 76.1% greater in summer than in autumn (*p* < 0.001). Overall higher variability in HCC was observed across all three groups during the winter and spring seasons compared to those in summer and autumn, as shown in Figure 4.

### 3.2 Group comparisons of cortisol concentrations within each season

Group comparisons within the seasons showed significant differences in HCC between the Out-1, Out-2 and Ind groups. In winter, HCC was 33.4% greater in the Out-1 group compared to Out-2 group (*p* = 0.040), and 45.6% greater than in the Ind group (*p* = 0.008). In contrast, no significant changes were observed between the Out-2 and Ind groups in winter. In spring, the only significant difference noted was between the Out-2 and Ind groups, with HCC in the Out-2 group being 26.7% lower than in the Ind group (*p* = 0.033). Summer comparisons showed significantly lower HCC in Out-1, which were 30.2 and 66.4% lower than in Out-2 (*p* = 0.007) and the Ind group (*p* < 0.001), respectively. In addition, Out-2 also had 51.8% lower HCC than the Ind group in this season (*p* < 0.001). In autumn, Out-1 had 52.0% lower HCC than Out-2 (*p* < 0.001) and 43.0% lower than Ind (*p* < 0.001). Conversely, no significant difference was found between Out-2 and Ind in autumn.

### 3.3 Cortisol concentration comparisons by sex

**Figure 5:**
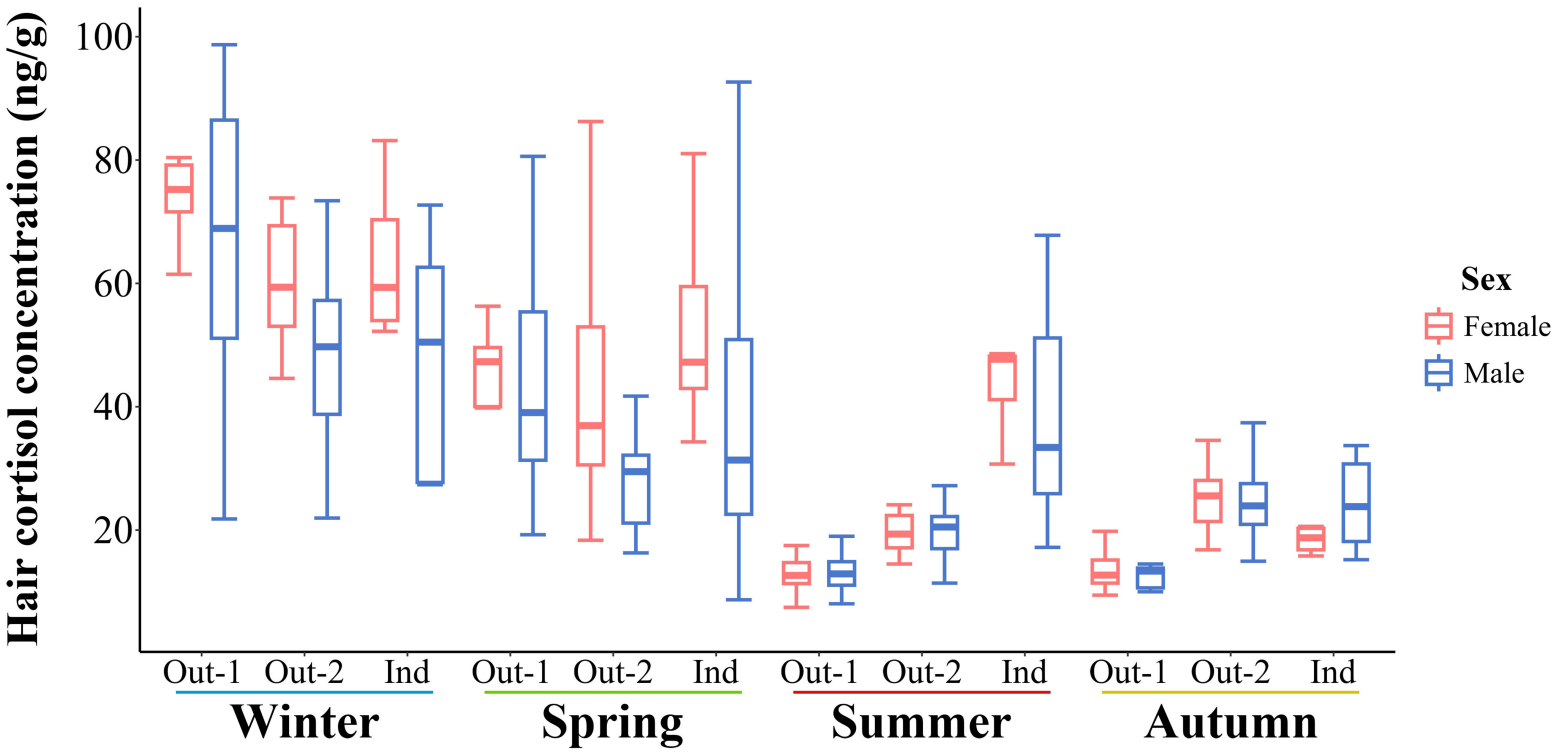
Boxplot representation of the seasonal hair cortisol concentrations in pigs by sex and group. Out-1, pigs, housed outdoors in all four seasons; Out-2, pigs, housed outdoors from spring to autumn; Ind, pigs, housed indoors in a barn in all four seasons.

There was no significant effect of sex on the predicted mean estimates of HCC. However, raw HCC measurements were more variable overall in male pigs for most measurements, as shown in Figure 5.

### 3.4 Recordings from the Postojna meteorological station

**Figure 6:**
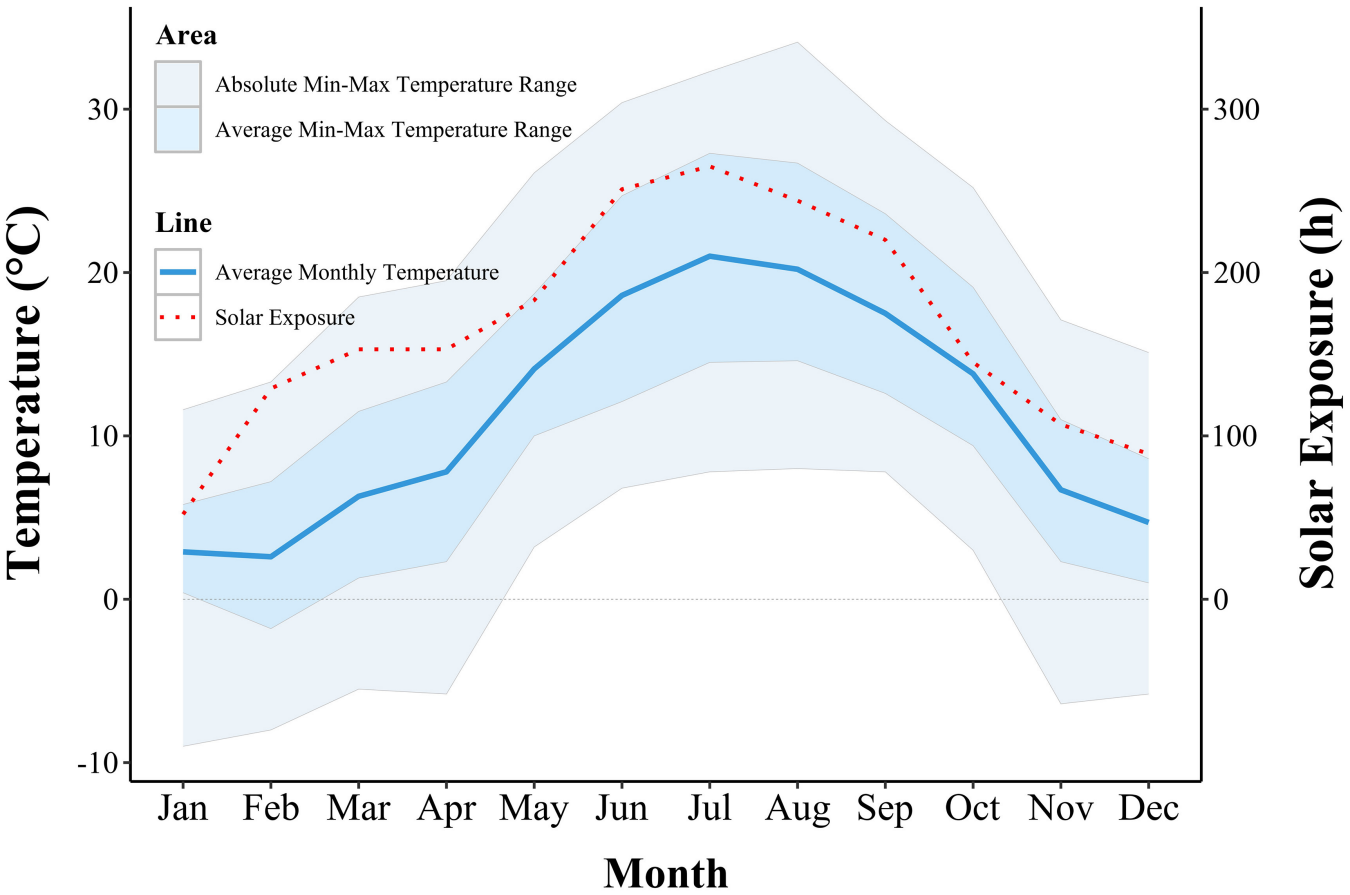
Graphical representation of ambient temperature and solar exposure recorded by the meteorological station, nearest to the farms.

Meteorological data for the area in which the farms were located is represented in Figure 6, showing the monthly temperature fluctuations and solar exposure during the year of sampling. The lowest average temperature of 2.6°C was measured in February and the lowest absolute minimal temperature of −9°C was recorded in January. Both the highest average temperature and the highest absolute maximal temperature were recorded in August and were 20.2 and 34.1°C, respectively. Solar exposure of 265 h in July was the longest, and 52 h of exposure was the shortest in January.

## 4 Discussion

In the present study, the HCC of 53 pigs from three different breeding systems were analyzed in all four seasons. The pigs in all three groups had the highest mean HCC in winter, combined with a very high variability in the measured values. Similarly, previous studies had reported elevated cortisol levels in winter in pigs (27) and cattle (28). Group Out-1 had the highest mean HCC, and no differences in HCC were detected between Out-2 and Ind groups. The pigs in Out-1 group were the only ones housed outdoors and on the sampling day, they were seen huddling on one side of the straw-bedded shelter. In contrast, Out-2 and Ind groups were housed indoors on a straw bedding. Pigs of the Out-1 group were 8 weeks old when they were relocated to the outdoor farm and 11 weeks old when the hair was sampled in winter sampling. The ideal ambient temperature for 11-week-old pigs is 20°C (29). During the first week after weaning, piglets require a lower critical temperature (the minimum body temperature that can be tolerated by an organism) of 26–28°C. Over the next 2 weeks, this temperature should be maintained at around 24°C, followed by a weekly decrease of 2–3°C until the slaughter (30). Therefore, the lower critical temperature for piglets aged 8–11 weeks is approximately 14–20°C. The average February temperature measured at the Postojna meteorological station, which was closest to our farms, was 2.6°C, with the average minimum temperature of −1.8°C and with absolute temperature minimum of −8°C. We can therefore assume that the pigs in the Out-1 group were experiencing higher discomfort due to the low temperatures outdoors, compared to the indoor-housed groups. The use of straw bedding can reduce the room temperature requirements of growing pigs (31).

The winter HCC assessment was conducted 3 weeks after the pigs' relocation. During this period, their hair grew between 4 and 9 mm, as pig hair typically grows at a rate of 5.3–12.0 mm per month (20). In the first weeks of their lives, all pigs were raised on the same organic farm, which maintained a high welfare standard. Therefore, any observed differences in HCC levels between the groups can be attributed to varying environmental conditions (indoor vs. outdoor). Additionally, elevated HCC levels across all groups may be explained by the stress associated with relocation, as studies indicate that transport and environmental factors are significant sources of stress for pigs (32, 33). We recorded the lowest mean HCC in summer and autumn. On the day of sampling in summer, we found sunburns in most pigs in the Out-1 and Out-2 groups. The severity of the sunburns was surprising, as all the pigs always had access to a shaded shelter. The average temperature in July was only 21°C, with an average maximum temperature of 27.3°C and absolute temperature maximum of 32.2°C. The sun exposure was also the highest in June, July and August with total monthly exposures of 251, 265, and 244 h, respectively. Sunburns can cause great discomfort and pain in pigs and lead to behavioral changes such as twitching and scratching (34), which were not observed during our visits. The presence of sunburns was also not obviously reflected in the HCC. We would expect an increase in HCC in pigs with sunburn in autumn, as the pigs' hair grows between 5.3 and 12.0 mm/month (20) and we sampled the hair every 3 months. However, the HCC did not differ significantly between summer and autumn.

We discovered a high variability in HCC as was already observed by many other authors (35–37). Measured HCC ranged from 7.5 to 100 ng/g. The hormonal response to stress depends on the animal's individual reactions. If an animal perceives an event as threatening, it will react regardless of whether the event is objectively threatening or not. Animals that react actively and try to cope with the threat have lower HCC than animals that react passively with avoidance or fear behavior (38, 39). Utilizing hair as a biomatrix for cortisol extraction offers a non-invasive method to assess long-term HPA axis activity (20). However, several external factors can cause variability in measured cortisol levels from collected samples, such as variations in collection site, hair color, hair segment (20), external contamination with feces, urine and saliva (23) and exposure to ultraviolet (UV) radiation (40–42). We tried to eliminate those influences by only collecting hair from withers area, which was in our experience usually clean and not contaminated with urine or feces. We used the whole hair, not just proximal or distal segments. UV light exposure plays a notable role in affecting hair cortisol levels. Prolonged exposure to sunlight can lead to the photodegradation of cortisol within the hair shaft, potentially resulting in lower HCC readings (42). However, in Otten et al. (41)'s study, HCC were affected by UV light in white, but not in black hair. Since our study focused on pigs reared outdoors, we could not control UV exposure's effect on HCC. Moreover, we analyzed a combination of black and white hair, so we cannot isolate any impact of hair color. The effect of UV light on HCC degradation may also explain why sunburns observed in the outdoor groups did not correspond with higher HCC. However, in Autumn, Out-2 group had higher HCC than Out-1 even with the same sun exposure.

In our study, the Ind group had significantly higher HCC in summer than the Out-1 and Out-2 groups, suggesting that environmental conditions affected pigs' adrenocortical response or that UV light majorly degraded cortisol in the hair of pigs in Out-1 and Out-2 groups. In a study by Perić et al. (43), cows kept indoors had significantly higher HCC from August to October. Overall, both Out-1 and Out-2 had their lowest HCC measured in the summer, and group Ind in autumn.

In our study, HCC decreased with the age of the pigs. Higher HCC in young animals after birth (<1 month old) compared to older animals have already been described in piglets by Heimbürge et al. (20). In the same study, a striking decrease in HCC was described in pigs from 2 to 10 weeks of age when the hair grown *in utero* was shed. They also described an increase in HCC after 10 weeks of age. In contrast, the pigs in our study were already 11 weeks old at the start of sampling in winter, and their HCC continued to decline in subsequent months, which does not align with these earlier findings. This suggests that the influence of age on HCC in our study was largely overshadowed by the pronounced effects of seasonality. Although the study design was not intended to assess the effect of aging, we expected that HCC in the indoor group would not follow the same pattern as in the outside groups, where the effect of seasons and UV light exposure was very clear. However, the effect of seasonality was less pronounced in the Ind group, compared to Out-1 and out-2, as expected.

We attribute the decrease in HCC over the year in all groups, the low levels of measured HCC even in the indoor group that was not greatly influenced by UV light exposure; and the lack of major group differences to the high welfare standards to which the pigs in our study were exposed. Straw bedding was used in both indoor and outdoor housing, as straw is one of the most optimal enrichment materials. It encourages exploratory behavior in pigs and is also edible, which has a positive effect on the pigs' digestion (44). The pigs remained in the same, stable group throughout the year. The floor area of the Ind group exceeded the minimum standards of 1 m^2^ per pig weighing more than 110 kg (22) and the Out-1 and Out-2 groups grazed on a large pasture. When living space is limited, the subordinate pigs cannot retreat from the dominant pigs and not all pigs always have access to feed and water. This creates a competitive environment in which aggression and social stress are often visible (33). A reduction in living space also negatively affects the pigs' ability to cope with environmental factors—in smaller, crowded spaces, pigs are more susceptible to heat stress (45).

We did not find significant differences in HCC between females and castrated males, which is in line with previous research (20, 46–48). However, Bergamin et al. (49) reported higher basal cortisol levels in sows, compared to castrated males.

A limitation of our study is the age of the pigs, which coincides with the seasons. Therefore, groups of different ages should be tested each season to reduce concurrent influences. We also did not measure the temperature on the farm, where group Ind was housed. To draw stronger conclusions on the effect of environment on HCC, we would need the indoor temperature measurements. To confirm if the peak in HCC in winter can be attributed to relocation stress or the temperature stress, a baseline HCC measurement on the primary farm before relocation should be made. Since UV light affects HCC in white but not black hair (39), separately sampling black and white hair could improve understanding of UV light's influence on HCC.

## 5 Conclusion

The present study highlights the complex interplay of factors influencing HCC in pigs across different breeding systems and seasons. A significant seasonal effect was found to differ depending on the breeding system. HCC were the highest and most variable for all groups in winter, and lowest with less variability in summer and autumn. Sex had no effect on HHC. The highest HCC was measured in Out-1 group in winter, as it was the only group housed outdoors at that time. The Ind group had significantly higher HCC in summer compared to the Out-1 and Out-2, which could be due to hair cortisol degradation by the UV light exposure in outdoor groups light. Our study suggests that the seasonality, environmental conditions and housing type influence the HCC of pigs. Our findings indicate that pigs kept indoors can have low stress levels if they are reared in an enriched environment with sufficient space, are allowed to express their natural behavior and are housed in stable groups. Combined organic systems, where the pigs are kept indoors during the colder months and outdoors for the rest of the year, seem to be the most suitable to ensure high welfare and low stress levels for Krškopolje pigs. However, for the results to be more conclusive, other parameters of pig welfare should also be assessed. Mixed organic breeding systems could be a good alternative to intensive pig production systems as they promote higher welfare levels, better health, and robustness of the pigs. To our knowledge, this is the first study in which the HCC of Krškopolje pigs has been measured.

## Data Availability

The original contributions presented in the study are included in the article/supplementary material, further inquiries can be directed to the corresponding author.
